# Muscle channelopathies and electrophysiological approach

**DOI:** 10.4103/0972-2327.40221

**Published:** 2008

**Authors:** Ajith Cherian, Neeraj N. Baheti, Abraham Kuruvilla

**Affiliations:** Department of Neurology, Sree Chitra Tirunal Institute for Medical Sciences and Technology, Trivandrum - 695 011, India

**Keywords:** Channelopathy, electromyographic, ion channel, myotonia, periodic paralysis

## Abstract

Myotonic syndromes and periodic paralyses are rare disorders of skeletal muscle characterized mainly by muscle stiffness or episodic attacks of weakness. Familial forms are caused by mutation in genes coding for skeletal muscle voltage ionic channels. Familial periodic paralysis and nondystrophic myotonias are disorders of skeletal muscle excitability caused by mutations in genes coding for voltage-gated ion channels. These diseases are characterized by episodic failure of motor activity due to muscle weakness (paralysis) or stiffness (myotonia). Clinical studies have identified two forms of periodic paralyses: hypokalemic periodic paralysis (hypoKPP) and hyperkalemic periodic paralysis (hyperKPP), based on changes in serum potassium levels during the attacks, and three distinct forms of myotonias: paramyotonia congenita (PC), potassium-aggravated myotonia (PAM), and myotonia congenita (MC). PC and PAM have been linked to missense mutations in the SCN4A gene, which encodes α subunit of the voltage-gated sodium channel, whereas MC is caused by mutations in the chloride channel gene (CLCN1). Exercise is known to trigger, aggravate, or relieve symptoms. Therefore, exercise can be used as a functional test in electromyography to improve the diagnosis of these muscle disorders. Abnormal changes in the compound muscle action potential can be disclosed using different exercise tests. Five electromyographic (EMG) patterns (I-V) that may be used in clinical practice as guides for molecular diagnosis are discussed.

Ion channels are membrane-bound proteins that perform key functions in virtually all human cells. Such channels are critically important for the normal functioning of the excitable tissues of the nervous system, such as muscle and brain. However, an increasing number of human diseases associated with dysfunctional ion channels are now being recognized. There is huge diversity among these ion channels. Some proteins are tissue specific, while others are widely distributed throughout the body. The resting membrane potential of excitable cells is entirely due to the presence of such ion channels.

These channels are integral to the fundamental processes of electrical signaling and excitation within the nervous system. Such neurological channelopathies are frequently genetically determined. In this article, the clinical, genetic, and electrophysiological aspects of this expanding group of muscle voltage-gated ionic channelopathies are reviewed.

## Classification of Ion Channels

Different classifications of ion channels exist. Table 1 is a list of genetic neurological channelopathies affecting muscle function according to ion type. Most voltage-gated ion channels have a similar basic structure. All have a large pore-forming subunit, which is located within the membrane. The pore-forming subunit (also called the α subunit) contains a central aqueous pore through which the relevant ion passes in response to voltage change-induced activation, also known as gating. In addition to the main α-subunit, it is common for voltage-gated ion channels to possess accessory subunits, which may be cytoplasmic or extracellular. Generally, these have an important function in modulating the basic conductance function of the α-subunits. The structural topology of all voltage-gated ion channels is remarkably conserved through evolution. Sodium channels are key players for membrane excitability, whereas calcium channels couple membrane excitation to muscle contraction. Chloride channels belong to a different gene family; they play an important role in stabilizing the resting membrane potential and help in membrane repolarization after excitation.

**Table 1:** Classification of ion channels

Channel	Muscle	Gene
Sodium channel	Hypokalemic periodic paralysis	SCN4A
	Hyperkalemic periodic paralysis	SCN4A
	Paramyotonia congenita	SCN4A
	Potassium-aggravated myotonia	SCN4A
Chloride channel	Myotonia congenita:	CLCN1
	Thomsen's (AD) and Becker's (AR)	
Calcium channel	Hypokalemic periodic paralysis	CACNA1S
Potassium channel	Andersen's syndrome	KCNJ2
	Hypokalemic periodic paralysis	KCNE3
	Hyperkalemic periodic paralysis	KCNE3

## Periodic Paralysis

### Hypokalemic periodic paralysis

Hypokalemic periodic paralysis (HypoKPP) is the most common form of periodic paralysis, with an incidence estimated to be 1 in 100,000. It is inherited in an autosomal dominant manner, but new mutations account for up to one-third of cases. Onset is from early childhood to the thirties. More than 60% of patients have their first attack by 16 years. Limbs and truncal musculature become weak, though the cranial nerves are spared. The attacks may be brought on by a period of exercise followed by rest or by carbohydrate loading enhanced by hypokalemia in serum. It is common for attacks to develop in the early hours of the morning, particularly if a large carbohydrate meal has been taken late the previous evening. Serum potassium is typically low at the onset of the attack but may normalize quickly. However, there is no correlation between serum potassium concentration and the severity of the weakness. The duration of the attack can vary from hours to days.

As in all forms of periodic paralyses the attack frequency tends to decline with age, but a fixed myopathy may develop. Rhabdomyolysis has been reported with certain CACNA1S mutations, mainly R528H. A few patients may have permanent residual weakness; they have a delayed onset, most often after 40 years of age, and often the disease progresses slowly over the years.

Limb myotonia never occurs in HypoKPP, though focal eyelid myotonia may occur. Cardiac arrhythmias are uncommon, as the ion channels mutated in HypoKPP and hyperkalemic periodic paralysis (HyperKPP) are not expressed in cardiac muscles.

The creatine kinase level is increased during attacks. Compound muscle action potentials (CMAPs) are reduced during attacks and decrease after sustained (5 min) maximal contraction. Provocative tests, such as potassium loading or induction of hypokalemia are unhelpful due to high false positivity; they are also potentially hazardous. DNA testing should be considered at an early stage. Muscle pathology is nonspecific and may show clear central vacuoles along with tubular aggregates. A comparison with HyperKPP is made in Table 2.

**Table 2:** Differentiating features of periodic paralyses

	Hyperkalaemic periodic paralysis	Hypokalaemic periodic paralysis
Inheritance	Autosomal dominant	Autosomal dominant
Age of onset	First decade; attacks increase in frequency and severity until age 50 when they decline	Second decade; the frequency of attacks is maximal between 15 and 35 years of age and then decreases with age
Exacerbating factors	Rest after exercise, cold, potassium loading, pregnancy, glucocorticoids, stress, ethanol, fasting (for example, early morning before breakfast)	Rest after exercise, cold, carbohydrate loading, menstruation
Distribution of weakness	Usually proximal and symmetric, flaccid; occasionally distal and asymmetric in exercised muscles	Paraparesis or tetraparesis; cardiac, respiratory, and facial musculature spared
Duration of attack	Minutes to hours. Attacks more frequent than in HypoKPP	Hours to days
Severity	Mild/moderate weakness; can be focal	Moderate/severe weakness
Additional features	May be associated with paraesthesiae before paralysis; Tendon reflexes are abnormally diminished or absent during the period of paralysis; many older patients develop a chronic progressive myopathy with permanent weakness that may go unrecognized; this mainly affects the pelvic girdle and proximal and distal lower limb muscles; myotonia or paramyotonia in nearly 50% of cases	A myopathic form results in a progressive fixed weakness, predominantly in the lower limbs, which occurs in about 25% of patients; this is independent of paralytic symptoms and may even be the sole manifestation of the disease; some mutations predispose to rhabdomyolysis
Relieved by	Carbohydrate intake, mild exercise	
Serum potassium	High, but can be normal	Low, rarely normal
EMG findings	Some have myotonic discharges	None
Acute treatment	Inhaled salbutamol	Oral potassium; if unable to take oral preparations, intravenous potassium can be given, diluted in mannitol
Preventative therapy	Acetazolamide, thiazide diuretics	Low sodium/high potassium diet; dichlorphenamide; acetazolamide
Ion channel gene	Sodium channel (SCN4A); potassium channel (KCNE3)	Calcium channel (CACNA1S); sodium channel (SCN4A); potassium channel (KCNE3)

Point mutations in three separate muscle channel genes may cause HypoKPP. The majority of cases harbor point mutations in the L-type calcium channel, CACNA1S(HOKPP 1). Far less frequently, mutations have been described in the muscle sodium channel SCN4A (HOKPP2) and in the potassium channel KCNE3 (HOKPP3). Mutations in the L-type calcium channel α1-subunit (dihydropyridine receptor) (CACNA1S),[1,2] located on chromosome 1q31, account for about 70% of the cases of HypoKPP.[3] All mutations are arginine substitutions in the voltage sensor (S4) of the channel protein. It remains unclear how mutations in CACNA1S, which does not have a major role in determining muscle membrane excitability, result in attacks of paralysis. The normal channel has two roles: (1) as a slow voltage-activated calcium channel and (2) excitation-contraction coupling with the ryanodine receptor. Mutated channels have enhanced inactivation, leading to a very small defect in the control of muscle resting membrane potential. Studies on intact intercostal muscle fibers showed that the resting membrane potential of the muscle fibers in patients with HypoKPP is depolarized by about 5-15 mV compared with the normal value of −85 mV. Reducing the external potassium concentration further depolarizes the fibers to −50 mV and renders them inexcitable.

There is reduced penetrance in females (50%) in contrast to the complete penetrance seen in males. R528H (domain II) and R1239H (domain IV) are two common mutations of the CACNA1S gene. About half of the women who have the R528H mutation and one-third of those with the R1239H mutation are asymptomatic. In contrast, more than 90% of males with a disease-causing mutation are symptomatic. Specific mutations appear to have discrete clinical features. R528H mutation of CACNA1S is common with later age of onset (14.5 years in males and 11.8 years in females) and presents with associated myalgias, while R1239H mutation has an earlier onset (10.4 years in males; 8.8 years in females). The other major group of HypoKPP is due to missense mutations in the voltage sensor of domain 2 of SCN4A (the same sodium channel affected in HyperKPP and paramyotonia congenita).[4] There is some genotype/phenotype correlation—for example, acetazolamide treatment is often deleterious in the R672G mutation of the SCN4A gene. Mutations in KCNE3 on chromosome 11q13-q14 associated with HypoKPP have been recently identified. It is thought to be due to a dominant missense mutation—Arg83His. Clinically two different phenotypes are seen due to the same genetic mutation. One presents with hypokalemic periodic paralysis, with the onset of paralytic weakness by the second decade of life. Here high carbohydrate meals do not act as a precipitant and recovery is facilitated by alcohol. The other presentation is with HyperKPP, having onset of weakness by the second year of life. Here episodes of weakness occur in sleep, last about 12 h, and improve with carbohydrate loading.

### HypoKPP associated with distal renal tubular acidosis

This condition is due to SLC4A1 mutation in chromosome 17q21-q22 its and inheritance is either autosomal recessive or dominant. SLC4A1 protein is a major glycoprotein of the erythrocyte membrane and mediates exchange of chloride and bicarbonate across the phospholipid bilayer. Other mutations of the same gene can cause spherocytosis and hemolytic anemia. HypoKPP due to this mutation is endemic in Northeast Thailand. Clinically, these patients have numerous episodes of periodic paralysis that may last for hours and may produce respiratory failure. Tendon reflexes are reduced or absent. Treatment is with potassium supplements and bicarbonate. Acetazolamide is not used as it exacerbates the acidosis. Laboratory tests show hyperchloremic acidosis and the urine has an alkaline pH with reduced ammonia. Skeletal evaluation shows growth failure and osteomalacia with pathologic fractures.

## Thyrotoxic HypoKPP

This is frequently seen in hyperthyroid patients. It is most common in Asians, among whom the incidence is about 2%. This disease is often underreported and the true incidence may be as much as 10% among hyperthyroid patients. It is relatively common in Native Americans also. Proclivity to the disease is thought to be inherited in autosomal dominant manner or it may be sporadic. Sporadic cases have shown mutation of KCNE3 (HOPP3). No CACN1AS or SCN4A mutations have yet been reported.[5] Male predominance (83-95%) is seen. The clinical onset is between 18-40 years. Proximal muscle weakness is associated with muscle discomfort and cramps and each episode lasts hours to days. The lower limbs are more involved than the upper limbs in a proximo-distal fashion. Weakness may selectively involve recently exercised muscles. Respiration and bulbar function may be affected during severe attacks. Attacks often occur in a random pattern without any obvious stimulus. Precipitating factors include carbohydrate challenge (with or without insulin), muscle cooling, rest after exercise, and thyroxine ingestion. Single or multiple episodes occur especially at night and is more common in the May-October (summer) season. Attacks may be aborted by exercise and abate when thyrotoxicosis resolves. Systemic thyrotoxicosis may be subclinical. Other features include sinus tachycardia, weight loss, nausea, and vomiting.

Thyrotoxic HypoKPP resolves without morbidity after treatment of the thyroid disease. Laboratory evaluation shows hypokalemia (usually <2.5 μmol/l), though occasionally potassium levels may be normal. Thyroid stimulating hormone (TSH) level is low and hypophosphatemia is seen. Electrodiagnostic study shows reduced CMAP amplitude during attacks. Treatment is by correction of thyrotoxicosis.

## Hyperkalemic Periodic Paralysis (HyperKPP)

HyperKPP is an autosomal dominant disorder with an estimated prevalence of 1:200,000. Patients experience attacks of either focal or generalized muscle weakness, often after exercise. Attacks may vary in severity from mild weakness to total paralysis. The duration of attacks is shorter than in HypoKPP; it typically lasts about an hour or two and may be associated with paresthesias. Attacks are provoked by exercise, potassium loading, cold environment, pregnancy, glucocorticoids, stress, ethanol, and fasting. Attacks are relieved by carbohydrate intake, mild exercise, salbutamol inhalation, and intravenous calcium gluconate.

The frequency of attacks declines with age, but patients often develop a fixed myopathy of variable severity.

It is notable that death is fortunately extremely rare in HyperKPP or HypoKPP. HyperKPP is caused by point mutations in the skeletal muscle sodium channel α-subunit, SCN4A (which is mutated in paramyotonia congenita).[6] These mutations lead to defective inactivation of the channel.[7] Some genotype/phenotype correlations can be made. For example, the most frequent point mutation, T704M, which occurs in 60% of cases, often leads to permanent late-onset muscle weakness. Another frequent mutation, I1592M, is often associated with myotonia in addition to paralysis. Laboratory evaluation during attacks shows high serum K^+^ (>4.5 mEq/l) and high urinary K^+^. Serum creatinine kinase (CK) levels vary from normal to 300 U/l. CMAP amplitudes are increased immediately after short (10 s) bouts of exercise and sustained (5 min) maximal contraction but are progressively reduced (by 40%) during rest, 20-40 min after the initial increment; (this is seen in 80% of the patients).

Many attacks are brief and do not need treatment. Acute attacks can be aborted by carbohydrate ingestion, salbutamol inhalation,[8] and intravenous calcium gluconate. Prophylactic therapy include acetazolamide and thiazide diuretics.[9]

### K^+^-sensitive periodic paralysis with cardiac arrhythmias (Andersen's syndrome)

Andersen's syndrome is an autosomal dominant, potassium-sensitive periodic paralysis with ventricular dysrhythmias and dysmorphic features.[10] The dysmorphic features are often subtle. They include low-set ears, hypertelorism, clinodactyly, and syndactyly. Bidirectional ventricular tachycardia is a frequent and potentially serious arrhythmia. From a practical point of view, this disorder should be considered in any case of periodic paralysis with arrhythmia. The resting electrocardiogram often shows bigeminy. It is now known that Andersen's syndrome is a cardioskeletal muscle channelopathy caused by mutations in a potassium channel termed Kir2.1. This inward-rectifying potassium channel is encoded by KCNJ2 on chromosome 17q23; the disease-causing mutations were first described in 2001.[11] The channel plays a part in cardiac and skeletal muscle membrane hyperpolarization and, interestingly, also has a role in skeletal bone precursor cell migration and fusion during development; hence, the triad of symptoms. Functional expression studies have shown loss of function due to a dominant negative effect on wild-type channel subunits, producing a reduced inwardly rectifying K^+^ current.[12] Intrafamilial variability and partial manifestation of the phenotype is common. Ventricular arrhythmias are common in females (81%), while periodic paralysis is seen more frequently in males (40%). Onset is between 2-18 years of age. The duration of an attack could range from 1 h to days. The proximal muscles are more involved than the distal. Precipitating factors include potassium and exercise; at times there may be no obvious precipitant. There is no myotonia, and permanent weakness is seen only occasionally. Sudden death can occur. Associated lesions include renal hypoplasia. Serum potassium during an attack may be high, low, or normal. In those with hypokalemia, oral potassium supplements may improve the weakness. In some families, increasing the plasma potassium concentration with acetazolamide improves arrhythmias at the expense of exacerbating the weakness.[13] Once the diagnosis is made, a detailed cardiac assessment is essential. However, the optimum management to prevent malignant arrhythmias is not certain. Currently, options include amiodarone and implantable cardioverter defibrillators.

### Practical approach to suspected periodic paralysis

A high index of suspicion, an accurate history, neurological examination during an attack, and measurement of serum potassium in a sample taken as early as possible after presentation are the key to making the diagnosis of periodic paralysis. HypoKPP can also occur in the context of hyperthyroidism (usually in Asians)[14] and hence thyroid function tests should also be measured. Other general medical causes of altered potassium concentrations should always be sought. Associated features that should lead to the suspicion of genetic periodic paralysis include dysmorphic features (Andersen's syndrome) and myotonia or paramyotonia. Electrophysiology of periodic paralysis may mimic axonal Guillian-Barré syndrome. If diagnostic uncertainty remains, referral to a specialist center should be considered.

**Figure 1:**
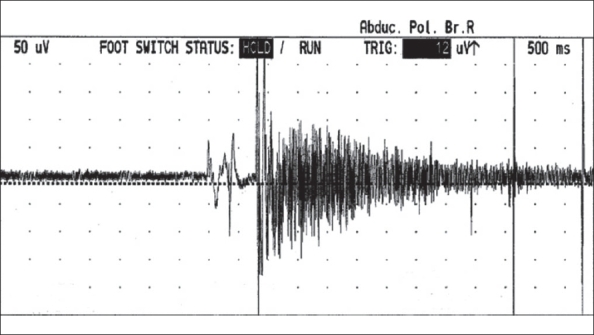
Myotonic discharges showing variation in amplitude and frequency within the discharge

It is important to define certain terms for further clarification:

- Myotonia refers to a disturbance in muscle relaxation after voluntary contraction or percussion. It improves with continued activity. Myotonic discharges are single-fiber action potentials whose waveform is that of positive sharp waves or fibrillations. Myotonic discharges in the EMG wax and wane in frequency (20-150 Hz) and amplitude (10 μv to 1 mv), producing a characteristic dive-bomber sound [Figure 1].
- Paramyotonia or paradoxical myotonia is stiffness (myotonia) that appears during exercise and worsens with continued activity.
- Neuromyotonia is a continuous muscle activity characterized by muscle rippling, muscle stiffness, and myotonia. The primary abnormality is in the nerve or nerve terminal. Neuromyotonic discharges are high-frequency (150-300 Hz) bursts of decrementing discharges of motor unit potentials that originate in motor axons and have an abrupt onset and offset. They result in characteristic high-pitched musical ‘pings’ over the loudspeaker. They can be spontaneous or may be initiated by needle movement, voluntary contraction of the muscle, or percussion of the nerve.
- Pseudomyotonia is an old term for neuromyotonia.
- Myoedema is an electrically silent, local bulge in the muscle that is induced by tapping; it is seen in myxedematous individuals.

## Paramyotonia Congenita

‘Paradoxical’ myotonia is stiffness (myotonia) that appears during exercise and worsens with continued activity. Electromyography at rest often shows some myotonia, although it is often less prominent than is seen in the other myotonias described below. Low temperatures often precipitate symptoms in these patients. Weakness or stiffness may occur alone. Myotonia is due to membrane depolarization caused by a mild slowing of sodium channel inactivation which is worse with cold and exercise. Myotonia is seen especially in the face, neck, and upper limbs. The onset of myotonia is in infancy.

Episodic weakness occurs due to muscle action potential blockade caused by very slow sodium channel inactivation. The association with HyperKPP is well known.

The weakness usually lasts a few minutes but may occasionally last for days. Onset of weakness is by adolescence or never. Some patients are worse after a potassium load.

Paramyotonia congenita is caused by mutations in the voltage-gated skeletal muscle sodium channel α-subunit (SCN4A) on chromosome 17q35.[15] Voltage-dependent activation of this channel results in an influx of sodium into the muscle fiber and is therefore responsible for the upstroke of the action potential. Rapid closure of this channel after activation is critical for muscle fiber repolarization. Paramyotonia congenita is inherited as a highly penetrant autosomal dominant trait. Mutations have been found throughout the gene.[16] Mild depolarization (>5 mV) produces repetitive discharges (myotonia), while more severe depolarization (>20 mV) produces weakness; either of these may occur as an isolated phenomenon.

### Potassium-aggravated myotonias

This is an umbrella term for several conditions that are due to mutations in the skeletal muscle voltage-gated sodium channel, SCN4A. Clinically, patients exhibit pure myotonia of variable severity, which can be particularly sensitive to potassium ingestion, with no associated weakness. Clinically, distinction from myotonia congenita, may be difficult. Various terms have been used to describe these disorders, which are summarized below.

#### Myotonia fluctuans:

This is characterized by mild myotonia that varies in severity from day to day; there is no weakness or cold sensitivity. Stiffness typically develops during rest after a period of exercise and lasts for approximately 1 h. It is exacerbated by potassium and depolarizing agents (for example, suxamethonium) and may interfere with respiration. The electromyogram shows myotonia, which increases after exercise.[17]

#### Myotonia permanens:

In this condition, patients experience severe continuous myotonia; it may interfere with respiration. There is often marked muscle hypertrophy, especially in the neck and shoulders.

#### Acetazolamide-responsive myotonia congenita:

This is characterized by muscle hypertrophy, myotonia, and myalgia; it is aggravated by potassium loading and improved by acetazolamide.[18] ‘Paradoxical’ myotonia is also seen.

## Myotonia Congenita

### Thomsen's disease:

Dr. Thomsen initially described this in his own family in 1876. Patients usually present between infancy and adulthood with mild myotonia, which may be constant or intermittent. Marked improvement in myotonia is noted with repeated exercise of a given muscle, which is called the ‘warm-up phenomenon.’ While 90% show myotonia on electromyography, only 50% have percussion myotonia on examination. There is usually normal power at rest, although some patients have proximal muscle weakness, which can present with functional difficulties such as when climbing stairs. Some patients have muscle hypertrophy, while others complain of myalgia. Electromyography shows myotonia with a distal predominance, which is present even in early childhood; the warm-up effect can be observed electrophysiologically.

Thomsen's disease is caused by mutations in a muscle voltage-gated chloride channel (CLCN1) located on chromosome 7q35.[19] It is transmitted as an autosomal dominant trait with variable penetrance, although 90% of affected individuals are symptomatic. This channel exists as a dimer; mutations may interfere with dimerization by exerting a dominant negative effect on the wild-type subunits.[20] Since chloride conductance is necessary to stabilize the high resting membrane potential of skeletal muscle, the loss of chloride conductance caused by mutations results in partial depolarization of the membrane, allowing increased excitability and myotonia.[21]

### Becker's disease:

The Becker form of myotonia congenita is more severe than Thomsen's disease and has an earlier age of onset. As in Thomsen's disease, there is myotonia with the warm-up phenomenon; but patients also have significant muscle hypertrophy, especially in the gluteal muscles. There may also be mild distal muscle weakness. Strength is normal initially, but there may be rapid decrease in power with short amounts of exercise, which returns to normal after further muscle contraction. Such transient weakness in Becker patients is more likely to happen after a period of rest; for example, after sitting for a while a patient may experience a transient lower limb weakness on standing. The electromyogram shows frequent myotonic discharges and the warm-up effect can be demonstrated. In contrast to Thomsen's disease, the motor units are frequently mildly myopathic. Becker's disease is also due to mutations in the muscle chloride channel (CLCN1),[19] hence the two forms of myotonia congenita are allelic. However, Becker's disease shows autosomal recessive inheritance. There is a male predominance, suggesting reduced penetrance or a milder clinical phenotype in females. Mutations have been found throughout the gene, with missense and nonsense mutations and deletions identified. Most patients are compound heterozygotes. Expression studies have indicated that the majority of mutations result in a loss of function of the chloride channel monomer.[20]

## Practical Management

Many patients with myotonia congenita do not require medication, but those that do usually respond well to mexilitine. Other antimyotonic agents can be considered, including phenytoin, but these are less effective.[22] Mexilitene causes use-dependent blockade of sodium channels and stops the production of repetitive runs of action potentials and hence reduces muscle stiffness. However, it can lead to arrhythmias, including torsades de pointes. Mexilitine treatment requires close monitoring with electrocardiography. A specific chloride channel opening agent would be the ideal therapy for such patients but such a drug has not been developed to date. Accurate genetic counseling is important, especially with regard to risks to offspring, and this is dependent on the availability of a precise DNA-based diagnosis.[23]

## Neuromuscular Junction

### Congenital myasthenic syndromes

There are several rare congenital myasthenic syndromes due to defects in the key processes that underlie efficient neuromuscular junction transmission. The commonest are mutations in the subunits of the postsynaptic acetylcholine receptor. These myasthenic syndromes may therefore be considered to be genetic ligand-gated channelopathies. Since this review is focused on genetic skeletal muscle voltage ionic channels, ligand-gated channelopathies will not be discussed further.

### Electrophysiological approach to channelopathies

Attempts were made in the past to correlate electrophysiological abnormalities with the phenotype. The main stumbling block was that in periodic paralysis the electrophysiological study is often normal in between the paralytic attacks. Various provocative methods were identified, which include short exercise test (SET), long exercise test (LET), high-frequency repetitive nerve stimulation (RNS). Fournier *et al*. identified the clinical phenotype and genotype of 51 patients and attempted to correlate it with EMG results. The utility of electrophysiology is as a guide towards various muscle channelopathy subgroups. Patients were divided into five subgroups based on the genotype/ phenotype, as follows:[24]

1. Paramyotonia congenita
2. Myotonia congenita
3. Potassium-aggravated myotonia
4. HyperKPP
5. HypoKPP

Two tests were done on these patient subgroups and the control arm:

- SET: This comprises isometric contraction of the abductor digiti minimi (ADM) lasting 10 s. CMAPs were recorded at baseline, 2 s immediately after the end of exercise, and then every 10 s for the next 50 s. The SET was repeated thrice with a 60-s break between trials.
- LET: Isometric contraction of the ADM lasting 5 min with brief (3-4 s) resting periods every 30-40 s to prevent ischemia. CMAPs were recorded 2 s immediately after cessation of exercise and then every minute for the first 5 min and, later, every 5 min for the next 45 min.

Control subjects after SET showed a slight and transient increase of CMAP amplitude by 5%, which returned to the preexercise value within 10 s. LET showed slightly reduced CMAP amplitude (by 6%) and increased duration (by 38%), which resulted in an increase in total area immediately after exercise with recovery within 1 min.[24]

**Figure 2:**
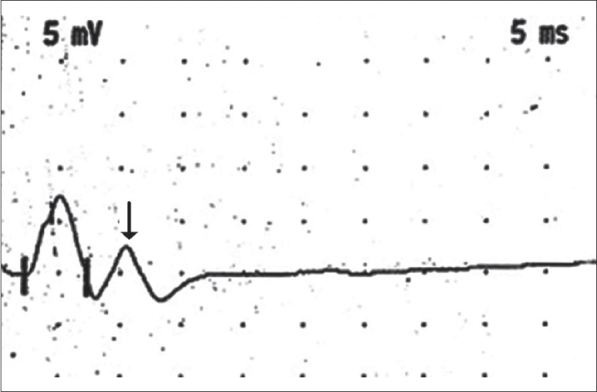
Single stimulation of the ulnar nerve revealing the ‘double hump pattern’; double peak of the compound muscle action potential (CMAP) in a patient with congenital myasthenia, which mimics PEMPs

In the type I pattern seen in the paramyotonia congenita group, abundant needle EMG myotonic discharges with postexercise myotonic potentials (PEMP) were seen in all patients. PEMPs are comparable to the repetitive discharges observed in neuromuscular junction disorders such as acetyl chlolinesterase deficiency and slow-channel syndrome[25] [Figure 2]. Both LET and repetition of SET lead to a long-lasting decrease in muscle electrical response. It may be due to exercise-induced sustained membrane depolarization leading to muscle electrical refractoriness. Type I pattern had a sensitivity of 100% for paramyotonia congenita.[24]

Type II pattern seen in myotonia congenita consisted of PEMPs in one-third of cases. SET after rest induced a transient decline of CMAP amplitude. Repeating the SET or LET reversed this block of muscle excitability. Sensitivity was 83%. Exercise-induced recovery of CMAP amplitude can be correlated clinically in majority of patients, in whom myotonia is relieved by exercise.

Type III pattern was seen in potassium-aggravated myotonia and was characterized by lack of PEMP and no change in CMAP amplitude after repeated SET or LET. It had a sensitivity of 63%.[24]

Type IV pattern was seen in HyperKPP. There is an increment in CMAPs immediately after both SET and LET and when short exercise was repeated. This correlates well with the observation that HyperKPP patients declare that repeated mild activity can improve their muscle strength and prevent or delay attacks of paralysis.

Type V pattern was seen in HypoKPP; SET did not produce any change in CMAPs. There was a late decline in CMAP response following LET, which speaks in favor of a reduced membrane excitability.[24]

## Conclusions

The muscle channelopathies are an important and expanding area within neurology. Electrophysiological exploration of patients with well-characterized mutations showed different patterns that may be related to distinct pathophysiological mechanisms and this can enable discrimination between different forms of periodic paralysis and myotonias. For many channelopathies, an accurate genetic diagnosis can be achieved. Genetic diagnosis is clearly important in order to allow accurate genetic counseling in appropriate families and will often inform treatment choices.
